# Publisher Correction: Thermodynamically controlled multiphase separation of heterogeneous liquid crystal colloids

**DOI:** 10.1038/s41467-023-41402-7

**Published:** 2023-09-08

**Authors:** Han Tao, Carlo Rigoni, Hailong Li, Antti Koistinen, Jaakko V. I. Timonen, Jiancheng Zhou, Eero Kontturi, Orlando J. Rojas, Guang Chu

**Affiliations:** 1 Department of Bioproducts and Biosystems, Aalto University School of Chemical Engineering, Vuorimiehentie 1, 02510 Espoo, Finland; 2 Department of Applied Physics, Aalto University School of Science, Puumiehenkuja 2, 02150 Espoo, Finland; 3 State Key Laboratory of Fine Chemicals, School of Chemical Engineering, Dalian University of Technology, Dalian, 116024 China; 4 School of Chemistry and Chemical Engineering, Southeast University, Nanjing, 211189 China; 5 Bioproducts Institute, Department of Chemical & Biological Engineering, Department of Chemistry and Department of Wood Science, The University of British Columbia, 2360 East Mall, Vancouver, BC V6T 1Z3 Canada

**Keywords:** Colloids, Colloids, Organic molecules in materials science

Correction to: *Nature Communications* 10.1038/s41467-023-41054-7, published online 29 August 2023

The original version of this Article contained an error in Fig. 3, in which a black rectangle overlays the multiphase region of the Figure 3c. This has been corrected in both the PDF and HTML versions of the Article.

