# Supplementary material for: Thermodynamically controlled multiphase separation of heterogeneous liquid crystal colloids
Source: Nat Commun. 2023 Aug 29;14:5277. doi: 10.1038/s41467-023-41054-7 (PMC10465492; doi:10.1038/s41467-023-41054-7)
Supplement: Supplementary file 1 — Supplementary Information [file 41467_2023_41054_MOESM1_ESM.pdf]

Supplementary Information

Thermodynamically Controlled Multiphase Separation of Heterogeneous Liquid Crystal Colloids

Han Tao[1], Carlo Rigoni[2], Hailong Li[3], Antti Koistinen[1], Jaakko V. I. Timonen[2], Jiancheng Zhou[4], Eero Kontturi[1,], Orlando J. Rojas[1,5,*] and Guang Chu[1,4,*]*

[1] Department of Bioproducts and Biosystems, Aalto University School of Chemical Engineering, Vuorimiehentie 1, 02510 Espoo, Finland

[2] Department of Applied Physics, Aalto University School of Science, Puumiehenkuja 2, 02150 Espoo, Finland

[3] State Key Laboratory of Fine Chemicals, School of Chemical Engineering, Dalian University of Technology, Dalian, 116024, China

[4] School of Chemistry and Chemical Engineering, Southeast University, Nanjing 211189, China

[5] Bioproducts Institute, Department of Chemical & Biological Engineering, Department of Chemistry and Department of Wood Science, 2360 East Mall, The University of British Columbia, Vancouver, BC V6T 1Z3, Canada

Corresponding Author:

Guang Chu: chuguang88@gmail.com

Eero Kontturi: eero.kontturi@aalto.fi

Orlando J. Rojas: orlando.rojas@aalto.fi, orlando.roja@ubc.ca

Supplementary Figures and Tables

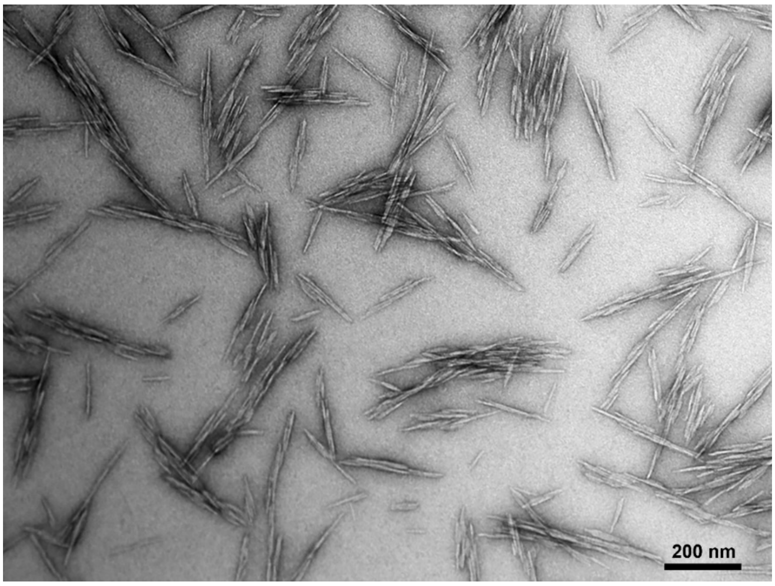

Supplementary Figure 1. TEM image of cellulose nanocrystals, showing individual CNCs with rod-like morphology.

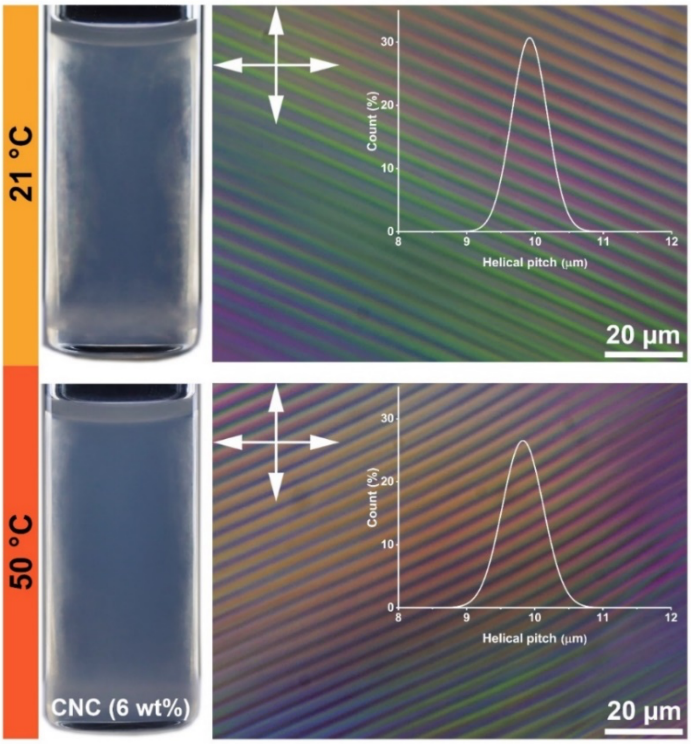

Supplementary Figure 2. Phase behavior of the liquid crystalline suspension of pure CNC with temperature-independent cholesteric assembly.

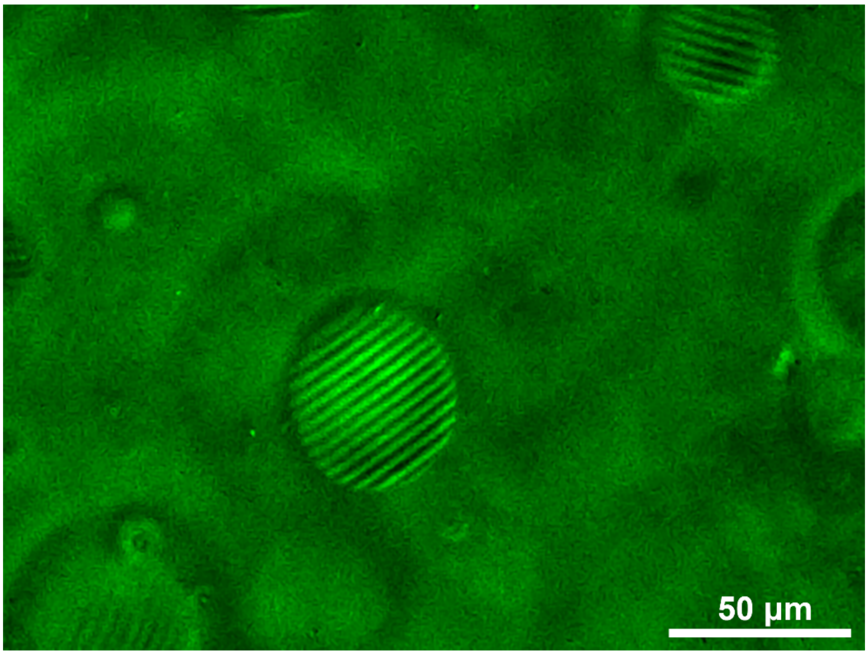

Supplementary Figure 3. Fluorescence image of the CNC-dextran mixture (6 wt%-4.25 wt%) with 0.01 wt% FITC-dextran as tracer, revealing strong fluorescent signals both in the cholesteric tactoid and its surrounding isotropic phase. These results suggest that dextran molecules were incorporated into the cholesteric liquid crystal phase of CNCs.

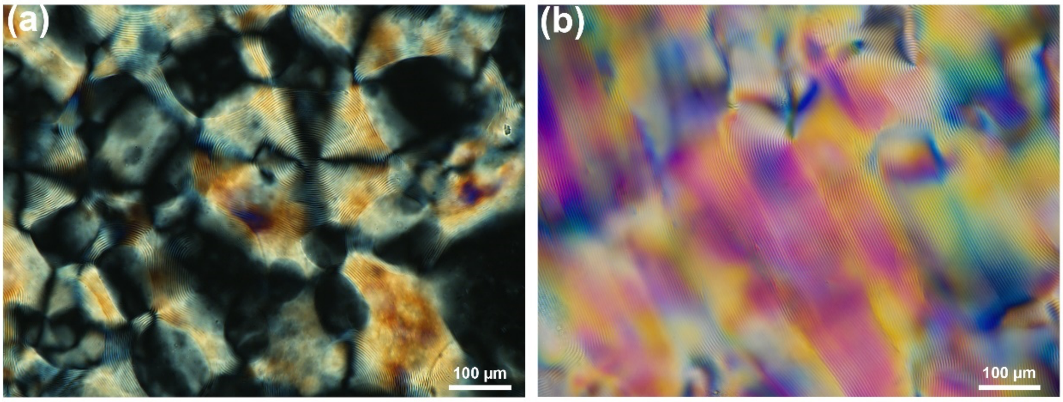

Supplementary Figure 4. POM images of the CNC-PEG (6 wt%-4.25 wt%) (a) and CNC-dextran (6 wt%-4.25 wt%) (b) mixtures, displaying a distinctive fingerprint texture typical of cholesteric organization. Specifically, vesicle-like structures are observed in the CNC-PEG mixture with isotropic interior and cholesteric surroundings, while the CNC-dextran mixture is homogeneous with a long-range ordered fingerprint texture.

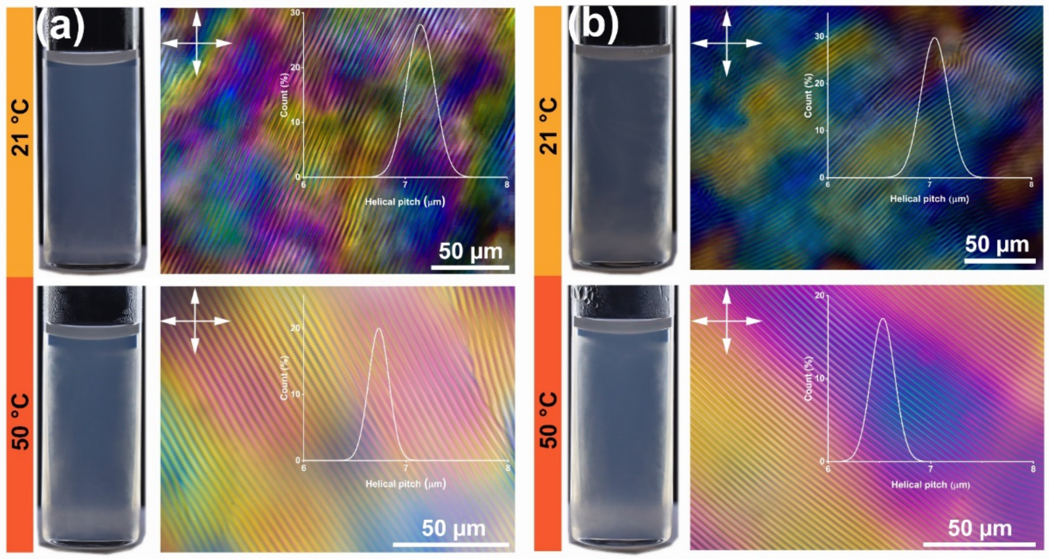

Supplementary Figure 5. CNC-dextran suspension with temperature sensitive phase behavior and cholesteric assembly. The composition of CNC-dextran is 6 wt%-3.5 wt% (a) and 6 wt%-4.25 wt% (b).

Supplementary Table 1. Helical pitch of cholesteric phase in the CNC-PEG-dextran heterogeneous colloidal suspension with various compositions after equilibrium at different temperature.

	CNC wt%	PEG wt%	Dextran wt%		Helical pitch µm
21 °C	6	0	0	Pure CNC	9.92 ± 0.26
50 °C					9.82 ± 0.21
21 °C	6	3.75	0	CNC/PEG	7.09 ± 0.14
50 °C					6.06 ± 0.11
21 °C	6	0	3.5	CNC/dextran	7.16 ± 0.16
50 °C					6.73 ± 0.09
21 °C	6	0	4.25	CNC/dextran	7.05 ± 0.13
50 °C					6.53 ± 0.11
21 °C	4	3.75	3.5	Dextran-rich cholesteric phase	9.05 ± 0.18
21 °C	6	3.75	3.5	PEG-rich cholesteric phase	10.19 ± 0.26
				Dextran-rich cholesteric phase	9.46 ± 0.26
50 °C				PEG-rich cholesteric phase	9.40 ± 0.27
21 °C	6	3.75	4.25	PEG-rich cholesteric phase	10.25 ± 0.42
				Dextran-rich cholesteric phase	8.03 ± 0.22
50 °C				PEG-rich cholesteric phase	7.14 ± 0.18
				Dextran-rich cholesteric phase	8.17 ± 0.26

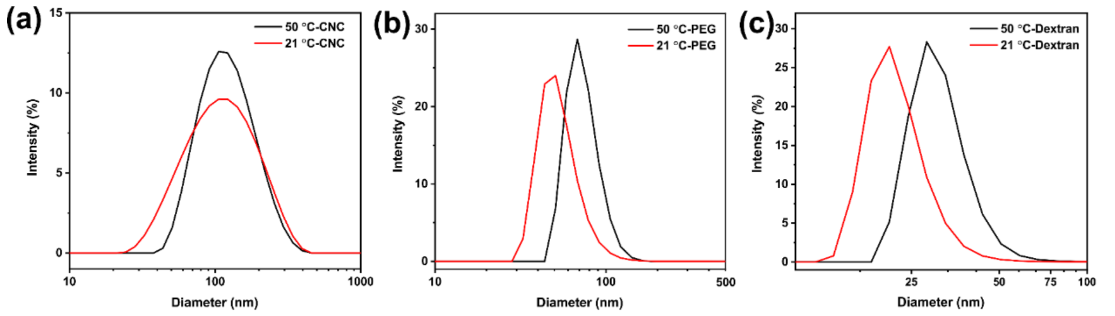

Supplementary Figure 6. Dynamic light scattering of (a) CNC, (b) PEG and (c) dextran aqueous suspension, respectively. These results demonstrate that the hydrodynamic size of PEG and dextran coils increased with temperature, whereas CNC remains constant.

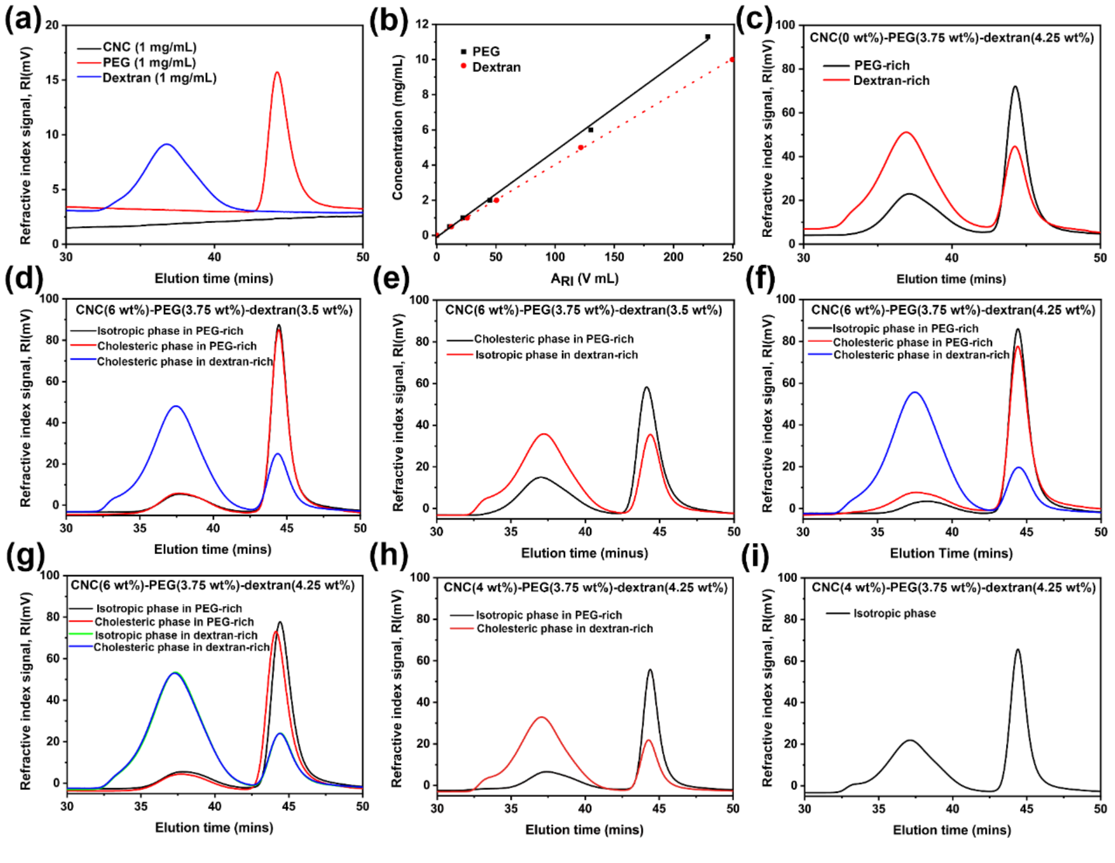

Supplementary Figure 7. (a) The size-exclusion chromatography for pure CNC, PEG and dextran with the injected concentration of 1 mg/mL, respectively. Dextran is eluted earlier with a peak at 36.95 minutes, while the peak for PEG appears later at 44.25 minutes. (b) The dependence of the RI peak area A_{RI} as a function of the polymer concentration. The size-exclusion chromatography of each phase in the CNC-PEG-dextran aqueous mixtures with different initial compositions: (c), (d), (f) and (h) were equilibrium under 21 °C, while (e), (g) and (i) were equilibrium under 50 °C, respectively.

Supplementary Table 2. Relative composition of each phase in the equilibrated CNC-PEG-dextran mixtures based on size-exclusion chromatography data.

	Initial Composition (wt%)				Density (g/cm^3)	Concentration (mg/mL)	
	PEG	Dextran	CNC			PEG	Dextran
21 °C	3.75	4.25	0	PEG-rich phase	1.025	40.82	31.36
				Dextran-rich phase	1.063	21.23	72.99
	3.75	3.5	6	PEG-rich isotropic phase	1.025	42.23	17.85
				PEG-rich cholesteric phase	1.175	50.00	13.57
				Dextran-rich cholesteric phase	1.242	17.13	81.20
	3.75	4.25	6	PEG-rich isotropic phase	1.032	44.90	8.28
				PEG-rich cholesteric phase	1.070	50.38	11.11
				Dextran-rich cholesteric phase	1.238	14.22	96.68
50 °C	3.75	3.5	6	PEG-rich cholesteric phase	1.193	43.23	26.83
				Dextran-rich isotropic phase	1.234	21.98	66.88
	3.75	4.25	6	PEG-rich isotropic phase	1.138	56.43	14.58
				PEG-rich cholesteric phase	1.208	54.36	15.75
				Dextran-rich isotropic phase	1.235	25.18	70.46
				Dextran-rich cholesteric phase	1.270	21.97	75.67

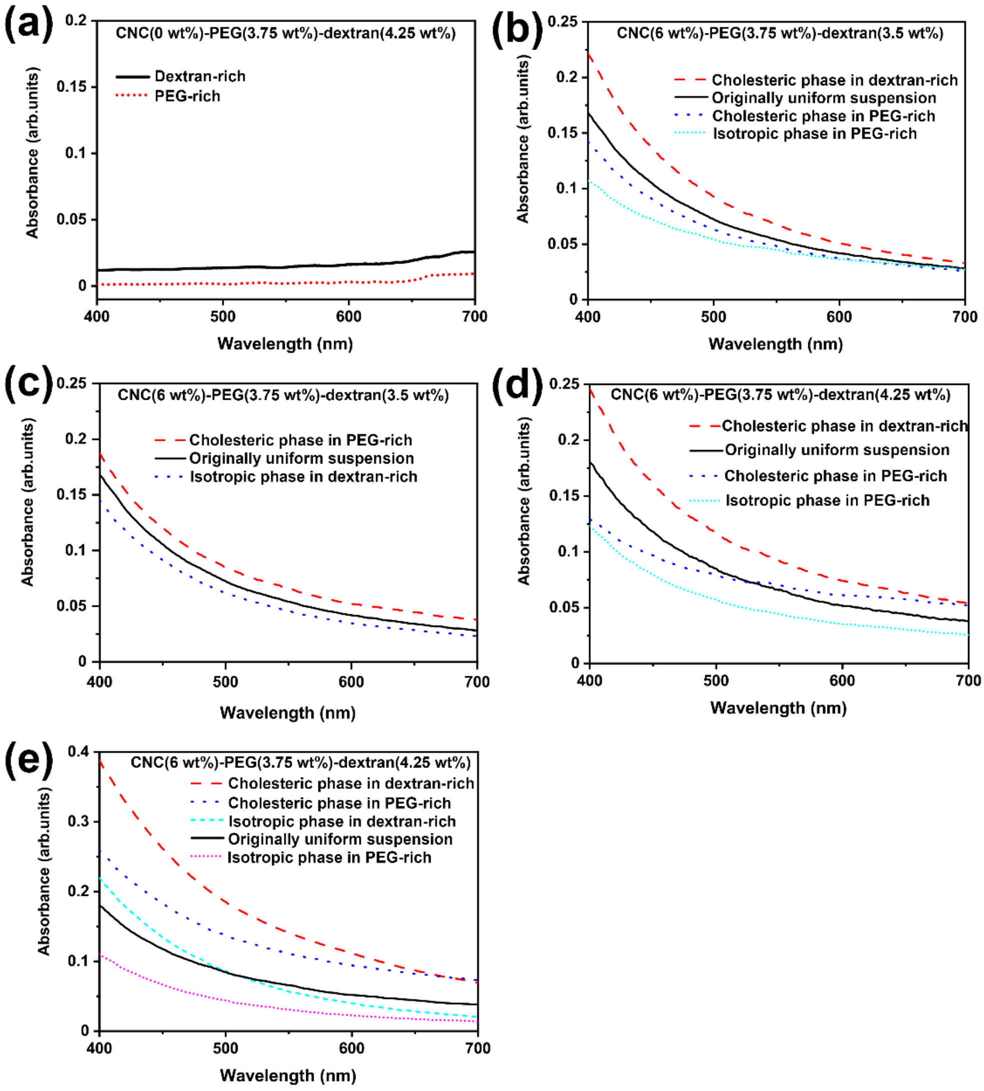

Supplementary Figure 8. UV-vis spectra of each phase in the CNC-PEG-dextran aqueous mixtures with different initial compositions: (a), (b) and (d) were equilibrium under 21 °C, while (c) and (e) were equilibrium under 50 °C, respectively.

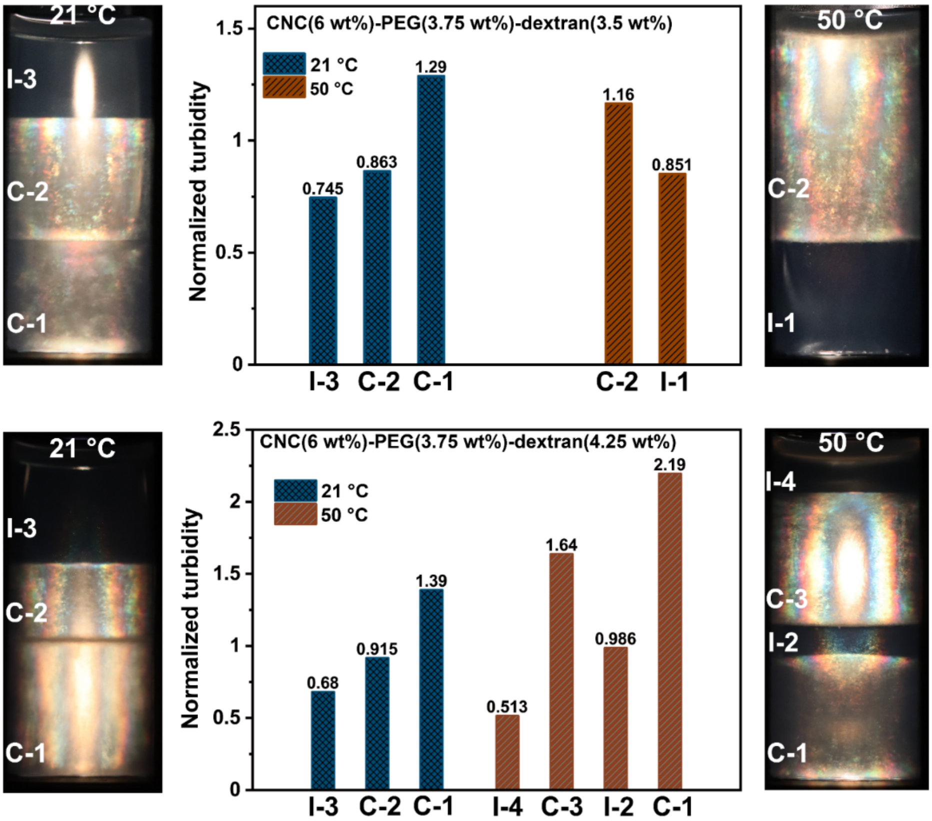

Supplementary Figure 9. Normalized turbidity calculated on basis of UV-vis measurements, which corresponds to the distribution of CNC in the CNC-PEG-dextran mixtures after reaching equilibrium at different temperatures.

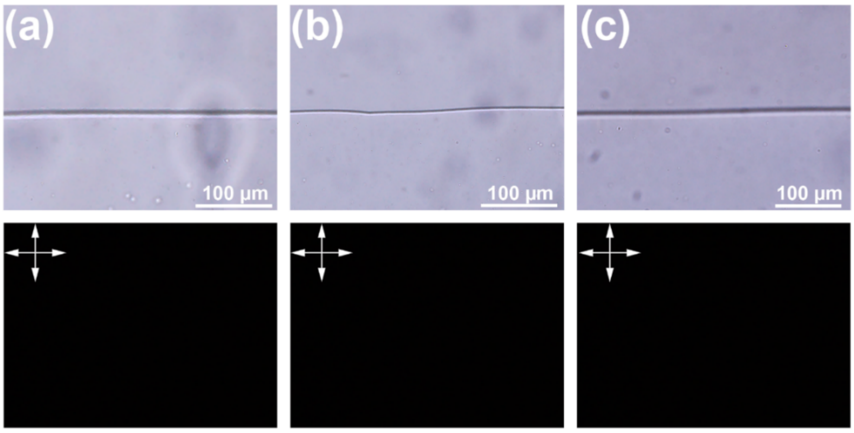

Supplementary Figure 10. Optical images of CNC-PEG-dextran three-component mixtures with particles content below the critical CNC concentration (4 wt%), showing two-phase stacking behavior without liquid crystalline ordering. The composition of PEG-dextran is fixed at 3.75 wt%-4.25 wt% and the CNC concentration increases from 1 wt% (a), 2 wt% (b) to 3 wt% (c).

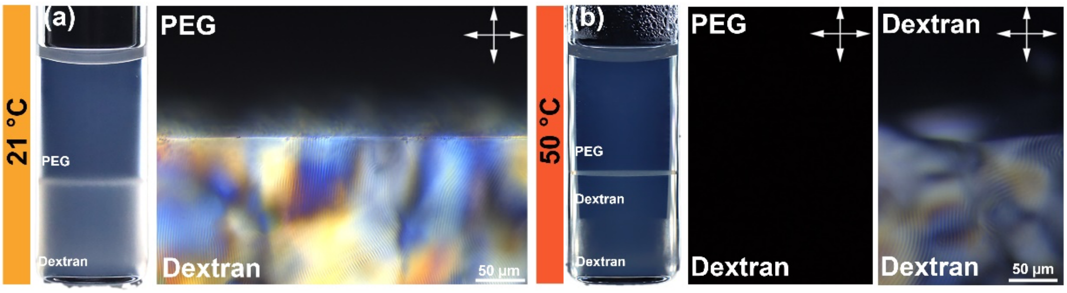

Supplementary Figure 11. Colloidal suspension of the CNC-PEG-dextran mixture with the composition of 4 wt%-3.75 wt%-4.25 wt% separated into two-phase stacking with an upper PEG-rich isotopic phase and a bottom dextran-rich cholesteric phase at 21 °C (a). While the mixture separated into three-phase stacking including an upper PEG-rich isotropic phase, a middle dextran-rich isotropic phase, and a bottom dextran-rich cholesteric phase at 50 °C (b).

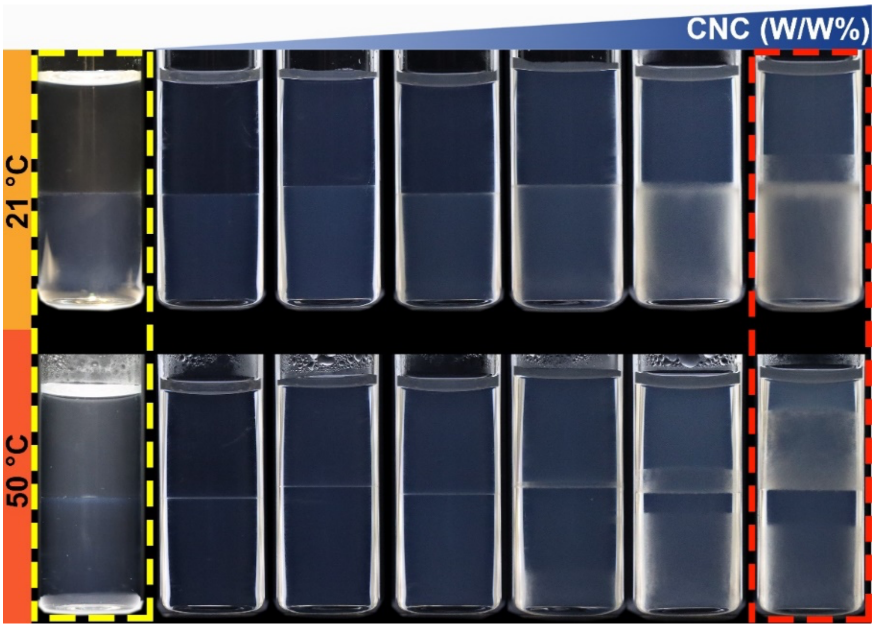

Supplementary Figure 12. The evolution of multiphase separation in the CNC-PEG-dextran mixtures with increasing CNC content from 0 (yellow box) to 6 wt% (red box) at different temperature as the composition of PEG-dextran is fixed at 3.75 wt%-5.5 wt%.

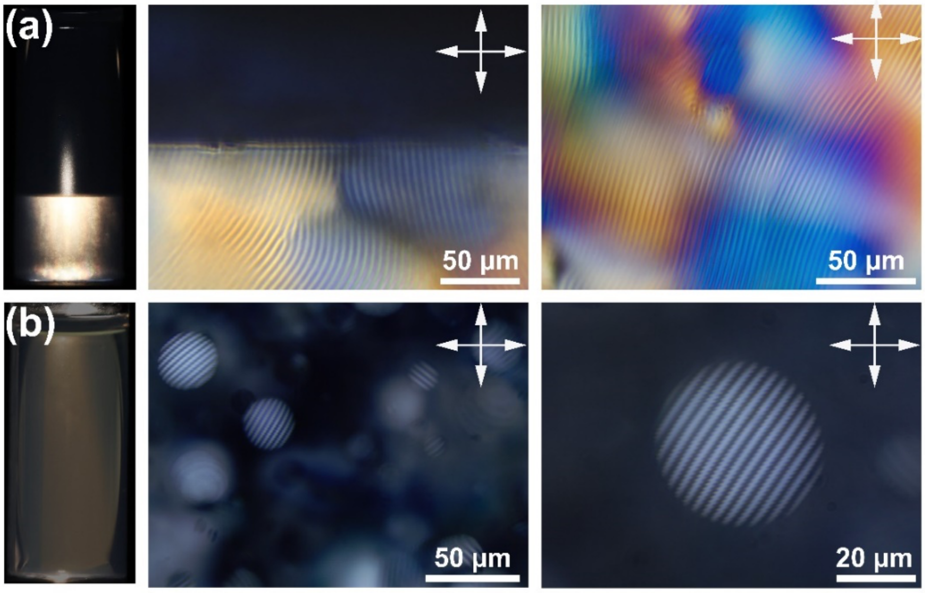

Supplementary Figure 13. Colloidal suspension of the CNC-PEG-dextran mixture with the composition of 4 wt%-3.75 wt%-3.5 wt% separated into two-phase stacking with an upper PEG-rich isotopic phase and a bottom dextran-rich cholesteric phase at 21 °C (a), while it remained isotropic with discrete CNC cholesteric tactoids when increased the temperature to 50 °C (b).

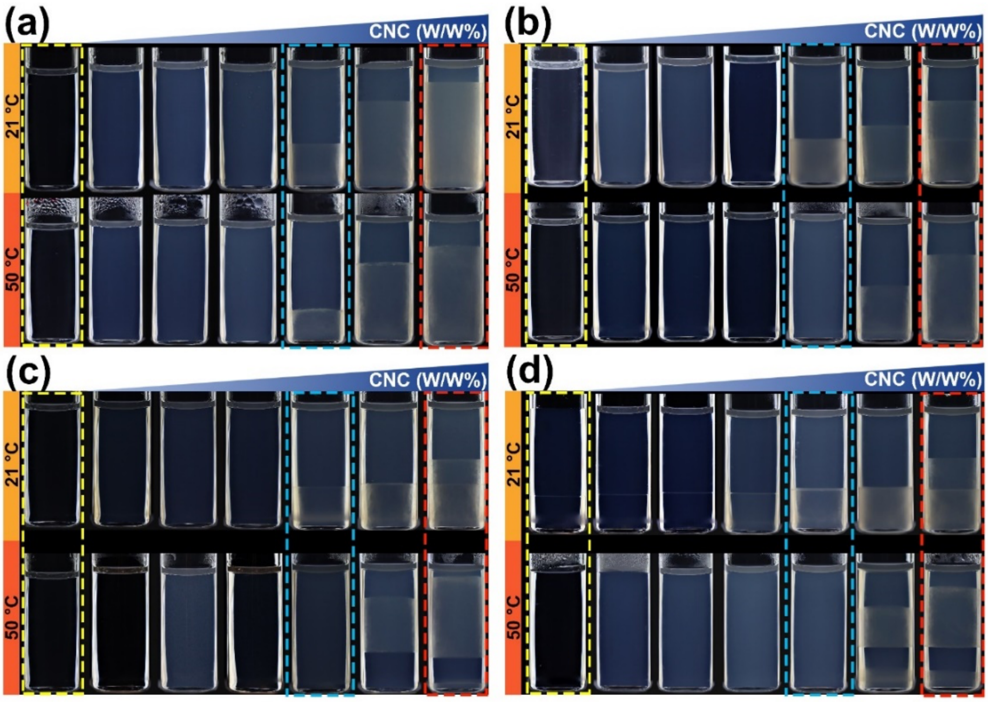

Supplementary Figure 14. The evolution of multiphase separation in the CNC-PEG-dextran mixtures with increasing CNC content from 0 (yellow box), 4 (blue box) to 6 wt% (red box) at different temperature. The composition of PEG-dextran is fixed at 3.5 wt%-3.5 wt% (a), 3.65 wt%-3.5 wt% (b), 3.85 wt%-3.5 wt% (c) and 4.0 wt%-3.5 wt% (d), respectively.

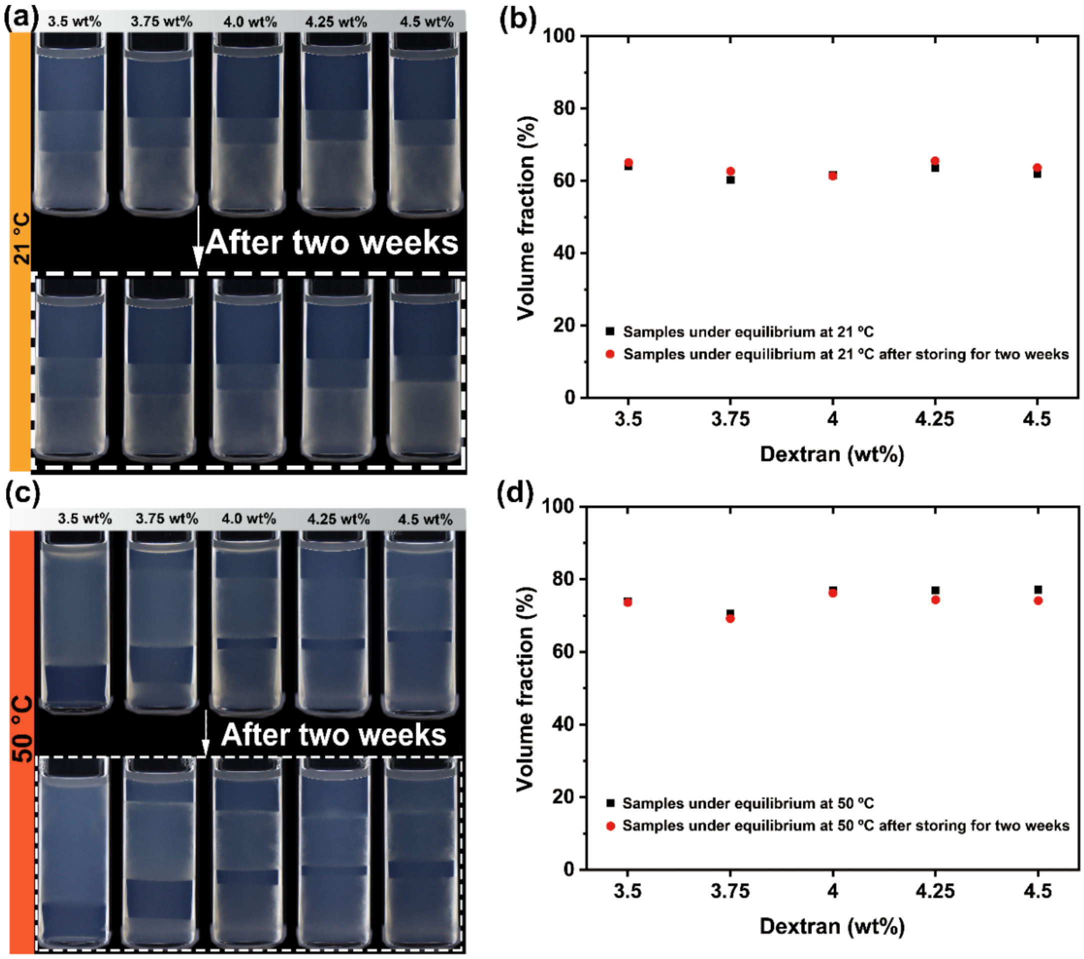

Supplementary Figure 15. The phase stability of the CNC-PEG-dextran mixtures that equilibrated under either 21 °C or 50 °C for two weeks. In these mixtures, the composition of CNC-PEG is fixed at 6 wt%-3.75 wt% with the dextran concentration increasing from 3.5 wt% to 4.5 wt%. Samples were equilibrated under 21 °C (a), and the variation of total volume fraction of the anisotropic phase as a function of dextran concentration before and after two weeks under 21 °C (b). Samples were equilibrated under 50 °C (c), and the variation of total volume fraction of the anisotropic phase as a function of dextran concentration before and after two weeks under 50 °C (d).

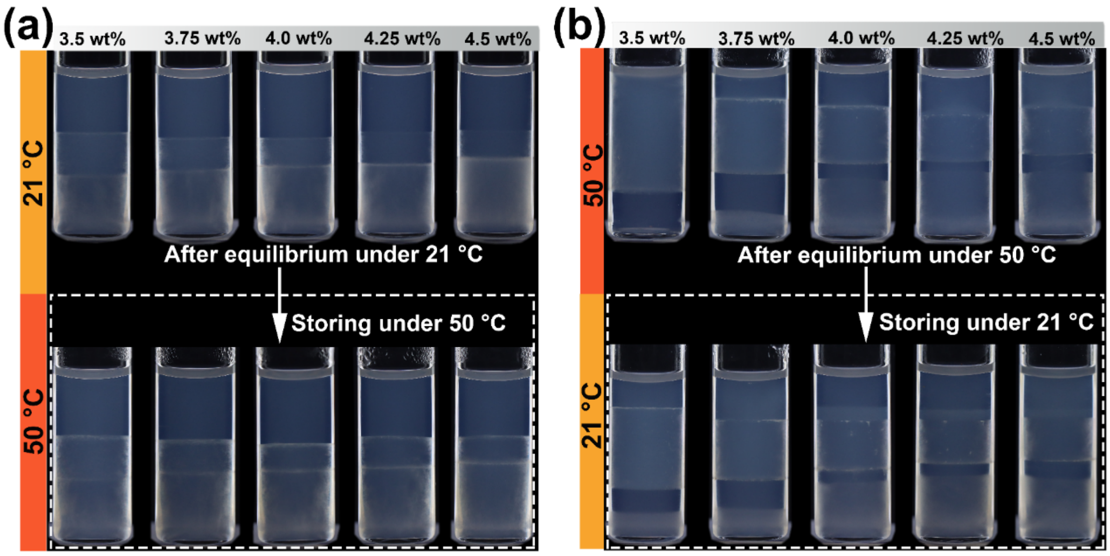

Supplementary Figure 16 (a), (b) The phase stability of CNC-PEG-dextran mixtures with switched equilibrated temperature.

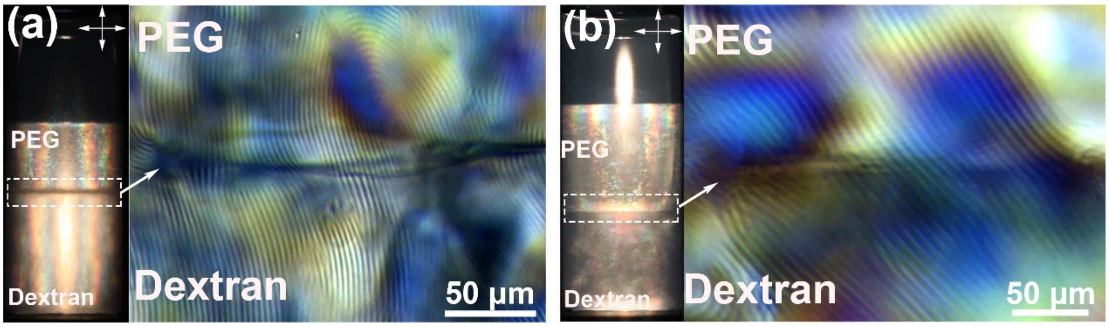

Supplementary Figure 17. High magnified POM images that focus on the PEG-dextran interface of the equilibrated four-component CNC-PEG-dextran aqueous mixture at 21 °C, showing continuous fingerprint texture propagates from the PEG-rich phase into the dextran-rich phase. The initial composition of the mixtures is 6 wt%-3.75 wt%-4.25 wt% for (a) and 6 wt%-3.75 wt%-3.5 wt% for (b).

Supplementary Table 3. The summary of interfacial tension for varying kind of phase boundaries.

	CNC wt%	PEG wt%	Dextran wt%		Interface	Interfacial tension μN/m
21 °C	6	0	0	Isotropic phase (I2)<br>Cholesteric phase (C1)	I2-C1	0.6
	0	3.75	4.25	PEG-rich phase (P)<br>Dextran-rich phase (D)	P-D	21.56
	4	3.75	3.5	PEG-rich isotropic phase (I2)<br>Dextran-rich cholesteric phase (C1)	I2-C1	3.63
	6	3.75	3.5	PEG-rich isotropic phase (I3)<br>PEG-rich cholesteric phase (C2)<br>Dextran-rich cholesteric phase(C1)	I3-C2<br>C2-C1	0.53<br>2.04
	6	3.75	4.25	PEG-rich isotropic phase (I3)<br>PEG-rich cholesteric phase (C2)<br>Dextran-rich cholesteric phase (C1)	I3-C2<br>C2-C1	0.74<br>9.37
50 °C	6	3.75	3.5	PEG-rich cholesteric phase (C2)<br>Dextran-rich cholesteric phase (I1)	C2-I1	0.13
	6	3.75	4.25	PEG-rich isotropic phase (I4)<br>PEG-rich cholesteric phase (C3)<br>Dextran-rich isotropic phase (I2)<br>Dextran-rich cholesteric phase (C1)	I4-C3<br>C3-I2<br>I2-C1	0.52<br>2.08<br>0.22

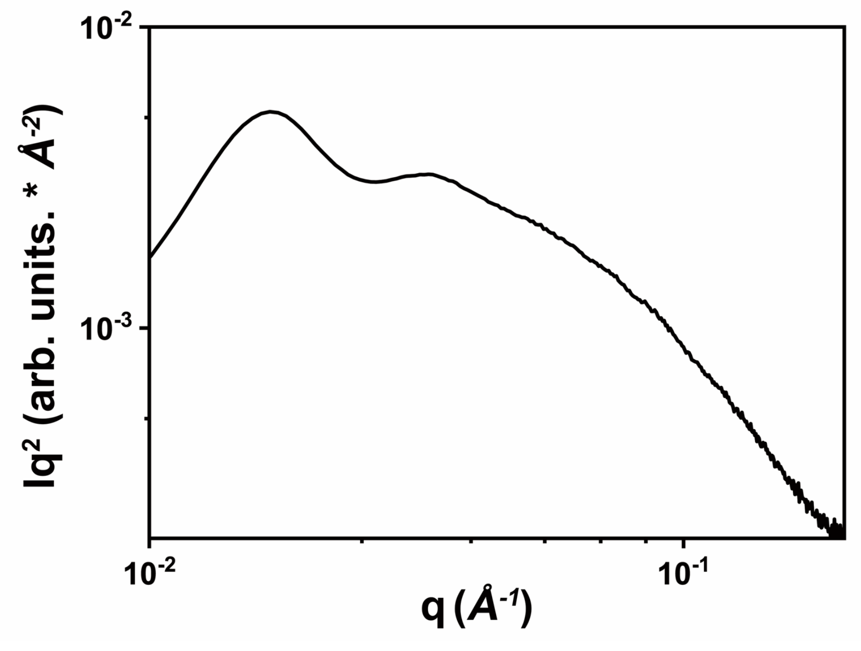

Supplementary Figure 18. SAXS data of the pure CNC cholesteric phase with the concentration of 6 wt%.

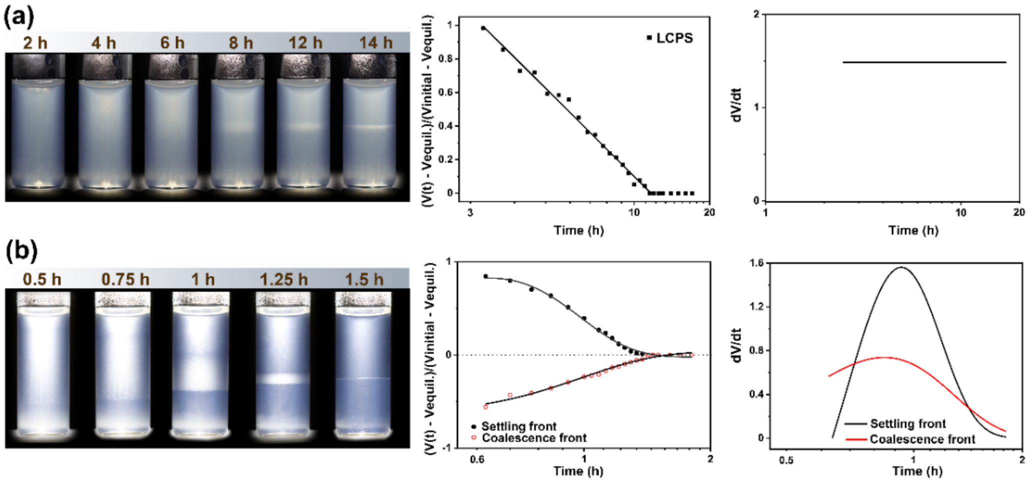

Supplementary Figure 19. (a) Time evolution of LCPS of the pure CNC suspension (6 wt%) and phase separation kinetic measured under 21 °C. (b) Time evolution of LLPS of a binary PEG-dextran mixture with the composition of 3.75 wt%-4.25 wt% and phase separation kinetic measured under 21 °C.

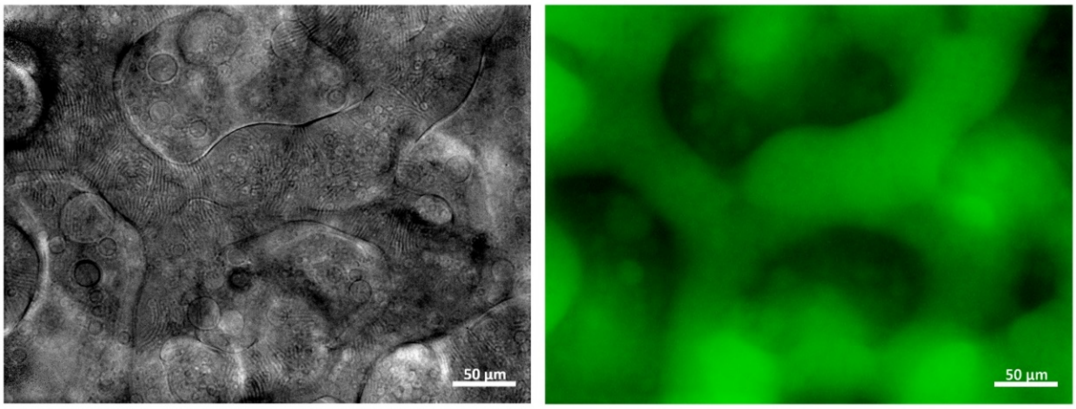

Supplementary Figure 20. Differential interference contrast and fluorescence images of the multiphase separation of the CNC-PEG-dextran mixture (6-3.75-4.25 (wt%) with the 0.01 wt% of FITC-dextran) in the elastic regime (0.5 h), showing the formation of network-like structure because of the mechanical balance of elastic force.

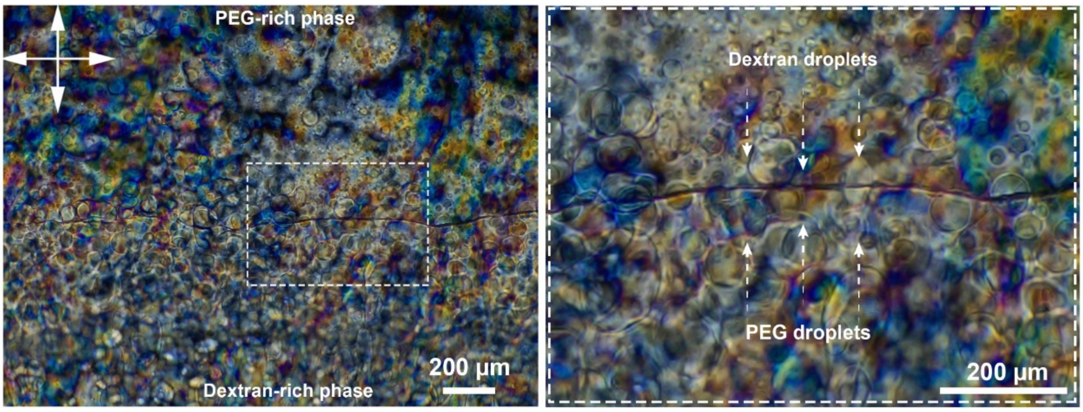

Supplementary Figure 21. POM images of the CNC-PEG-dextran mixture with the composition of 6 wt%-3.75 wt%-4.25 wt% focus on PEG-dextran phase boundary under 21 °C, showing that PEG droplets in the dextran-rich phase moved upward and dextran droplets in the PEG-rich phase sedimented downward.

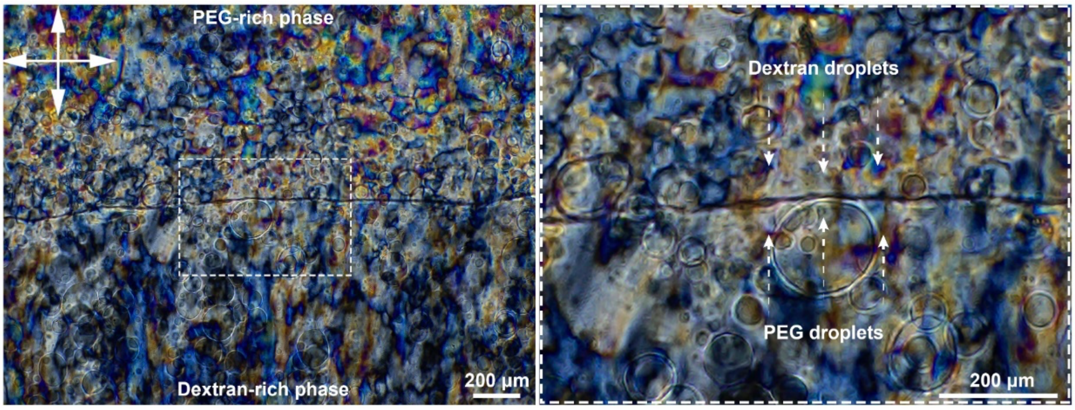

Supplementary Figure 22. POM images of the CNC-PEG-dextran mixture with the composition of 6 wt%-3.75 wt%-4.25 wt% focus on PEG-dextran phase boundary under 50 °C, showing that PEG droplets in the dextran-rich phase moved upward and dextran droplets in the PEG-rich phase sedimented downward.

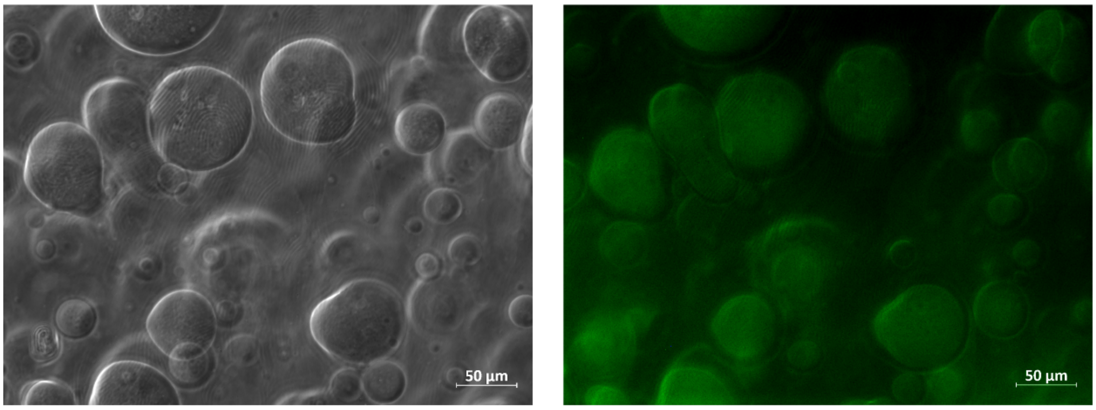

Supplementary Figure 23. Differential interference contrast and fluorescence images of the multiphase separation of the CNC-PEG-dextran mixture (6-3.75-4.25 (wt%) with the 0.01 wt% of FITC-dextran) in the hydrodynamic regime (1.5 h), showing the spherical droplets that were relaxed from the network-like patterns.

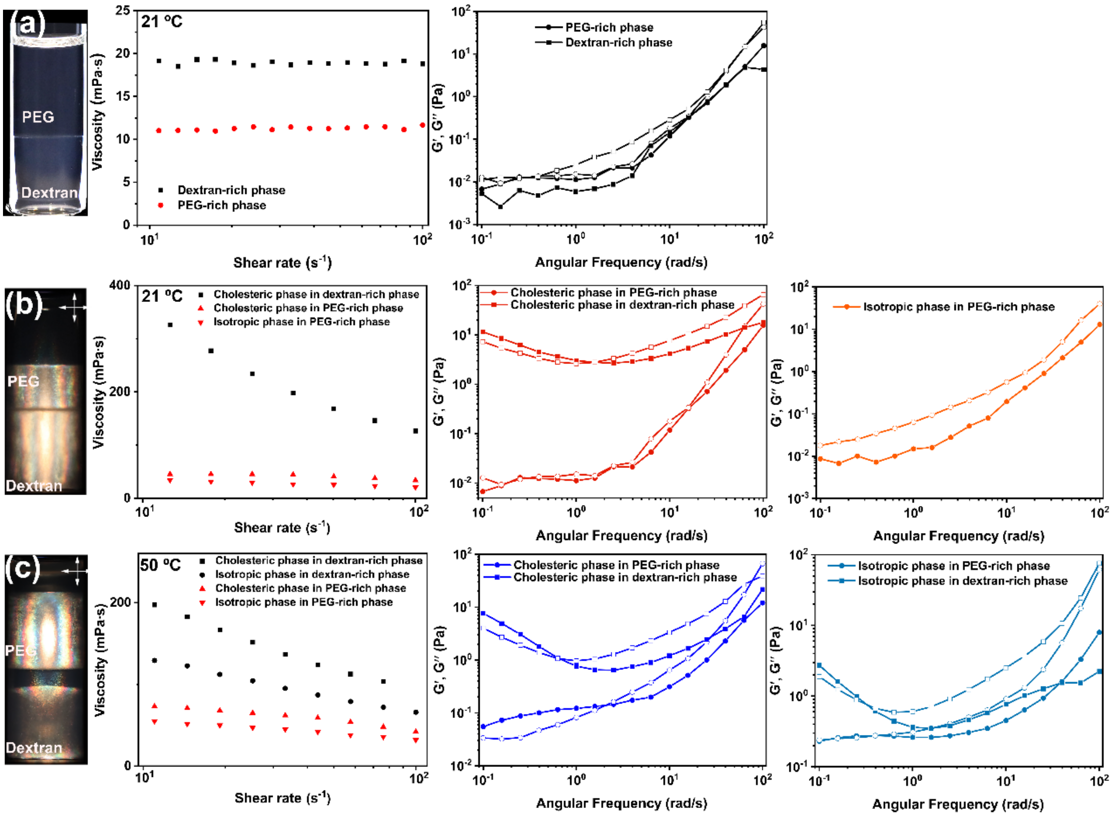

Supplementary Figure 24. (a)-(c) Viscosity profile and viscoelastic behavior (storage modulus G′ with filled symbols and loss modulus G″ with open symbols) for the PEG-dextran system (3.75 wt%-4.25 wt%) and CNC-PEG-dextran aqueous mixture (6.0 wt%-3.75 wt%-4.25 wt%) at different temperature.

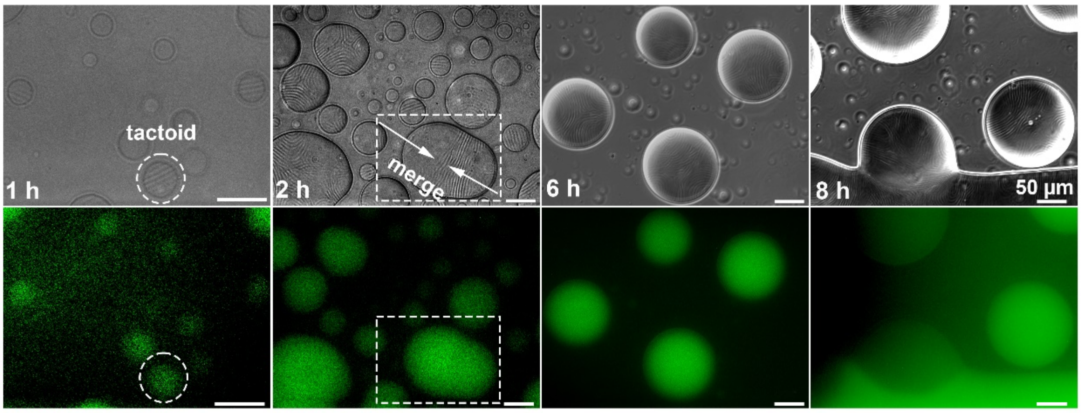

Supplementary Figure 25. Differential interference contrast and fluorescence images of the multiphase separation through the nucleation and grow process in the CNC-PEG-dextran mixture (4-3.75-3.5 (wt%) with the 0.01 wt% of FITC-dextran). The FITC-dextran molecules are enriched inside the cholesteric tactoids and merge into larger droplets, and eventually fuse into bulk dextran-rich cholesteric phase.

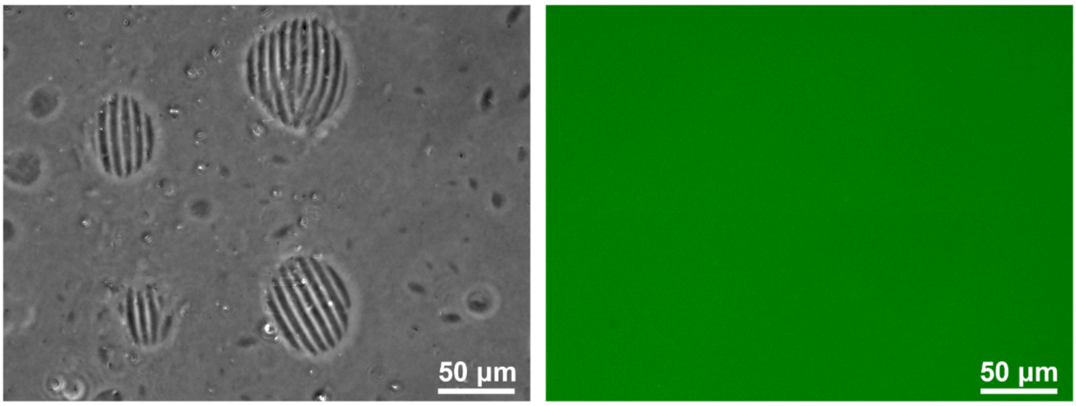

Supplementary Figure 26. Differential interference contrast (left) and fluorescence (right) images of the CNC-PEG-dextran homogenous mixture at 50 °C. The CNC-PEG-dextran composition in suspension is 4 wt%-3.75 wt%-3.5 wt% with the 0.01 wt% of FITC-dextran.

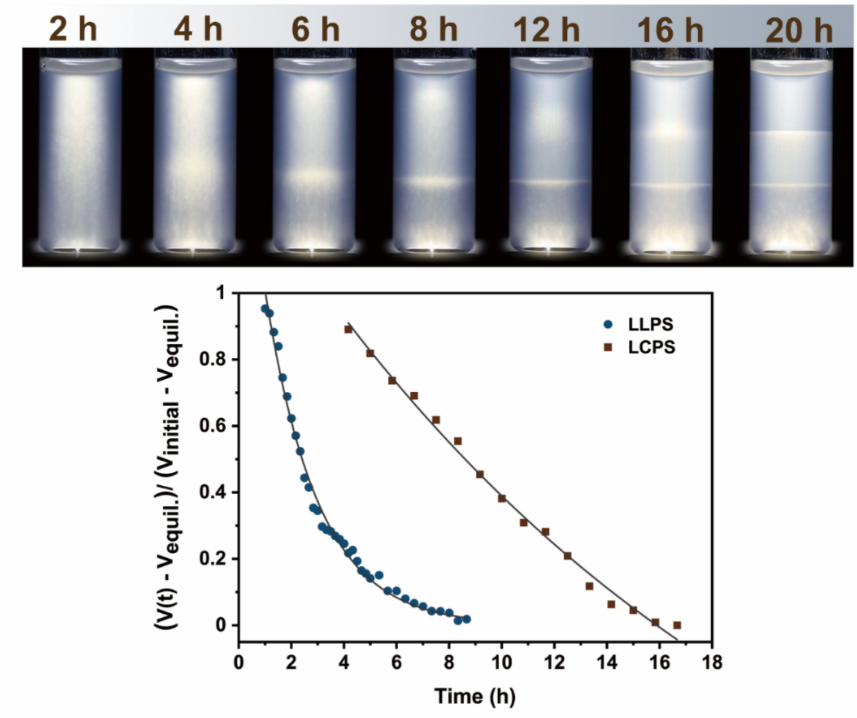

Supplementary Figure 27. Time evolution of the multiphase separation of the CNC-PEG-dextran mixture (6-3.75-3.5 (wt%)) and multiphase separation kinetic measured under 21 °C.

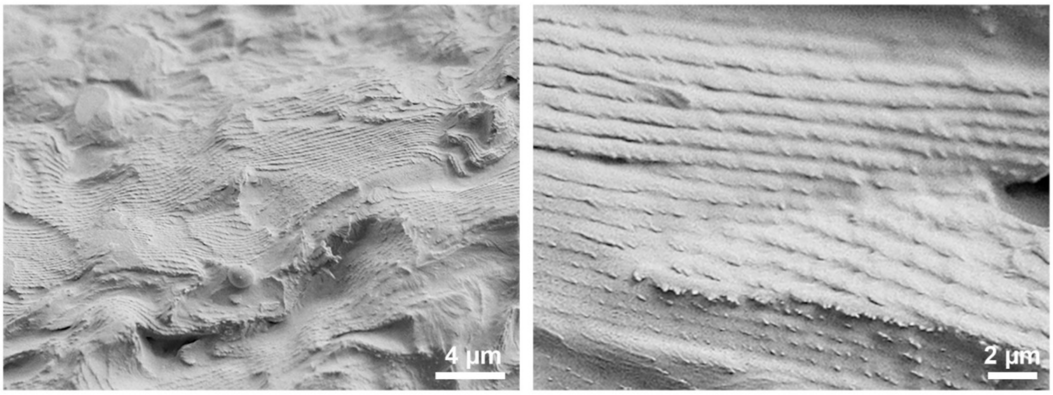

Supplementary Figure 28. SEM images of PEG-rich region that display denser and periodic layered structure.

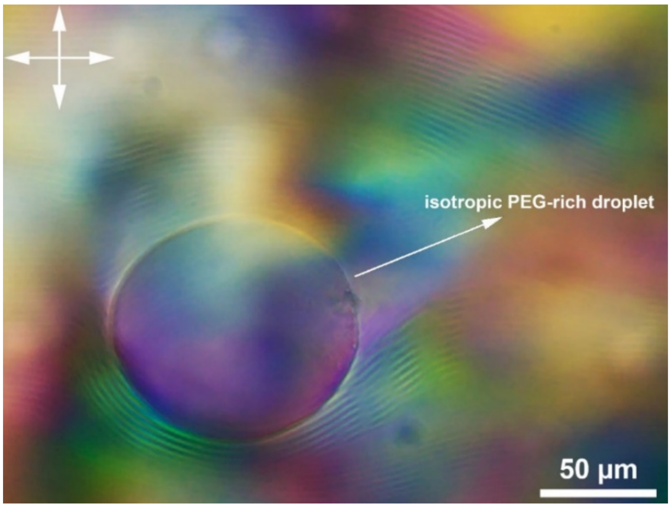

Supplementary Figure 29. POM image of an isotropic PEG-rich droplet (negative tactoid) surrounded by the dextran-rich cholesteric phase in the CNC-PEG-dextran mixture (6-3.75-3.5 (wt%)) under 21 °C.
