# Supplementary material for: Thermodynamically controlled multiphase separation of heterogeneous liquid crystal colloids
Source: Nat Commun. 2023 Aug 29;14:5277. doi: 10.1038/s41467-023-41054-7 (PMC10465492; doi:10.1038/s41467-023-41054-7)
Supplement: Supplementary file 3 — Description of Additional Supplementary Files [file 41467_2023_41054_MOESM3_ESM.pdf]

Description of Additional Supplementary Files

Supplementary Movie 1. Time-lapse video of the multiphase separation toward to three-phase stacking under 21 °C. The composition of the CNC-PEG-dextran mixture is 6 wt%-3.75 wt%-4.25 wt%.

Supplementary Movie 2. Time-lapse video of the multiphase separation with four-phase stacking under 50 °C. The composition of CNC-PEG-dextran mixture is 6 wt%-3.75 wt%-4.25 wt%.

Supplementary Movie 3. Time-lapse video that tracks LLPS process of PEG-dextran solution (3.75 wt%-4.25%) under 21 °C.

Supplementary Movie 4. Time-lapse video that tracks the LCPS process of pure CNC suspension with the concentration of 6 wt% under 21 °C.

Supplementary Movie 5. Time-lapse video with a coupled LLPS-LCPS multiphase separation process in the CNC-PEG-dextran mixture (4 wt%-3.75 wt%-3.5 wt%) under 21 °C.

Supplementary Movie 6. Time-lapse video showing the evolution of reverse phase separation of the CNC-PEG-dextran mixture (6 wt%-3.75 wt%-3.5 wt) toward two-phase stacking under 50 °C.

Supplementary Movie 7. Time-lapse video showing the evolution of multiphase separation of the CNC-PEG-dextran mixture (6 wt%-3.75 wt%-3.5 wt%) toward three-phase stacking under 21 °C.
