# Supplementary figures and images for: Thermodynamically controlled multiphase separation of heterogeneous liquid crystal colloids
Source: Nat Commun. 2023 Aug 29;14:5277. doi: 10.1038/s41467-023-41054-7 (PMC10465492; doi:10.1038/s41467-023-41054-7)

## Slide 1
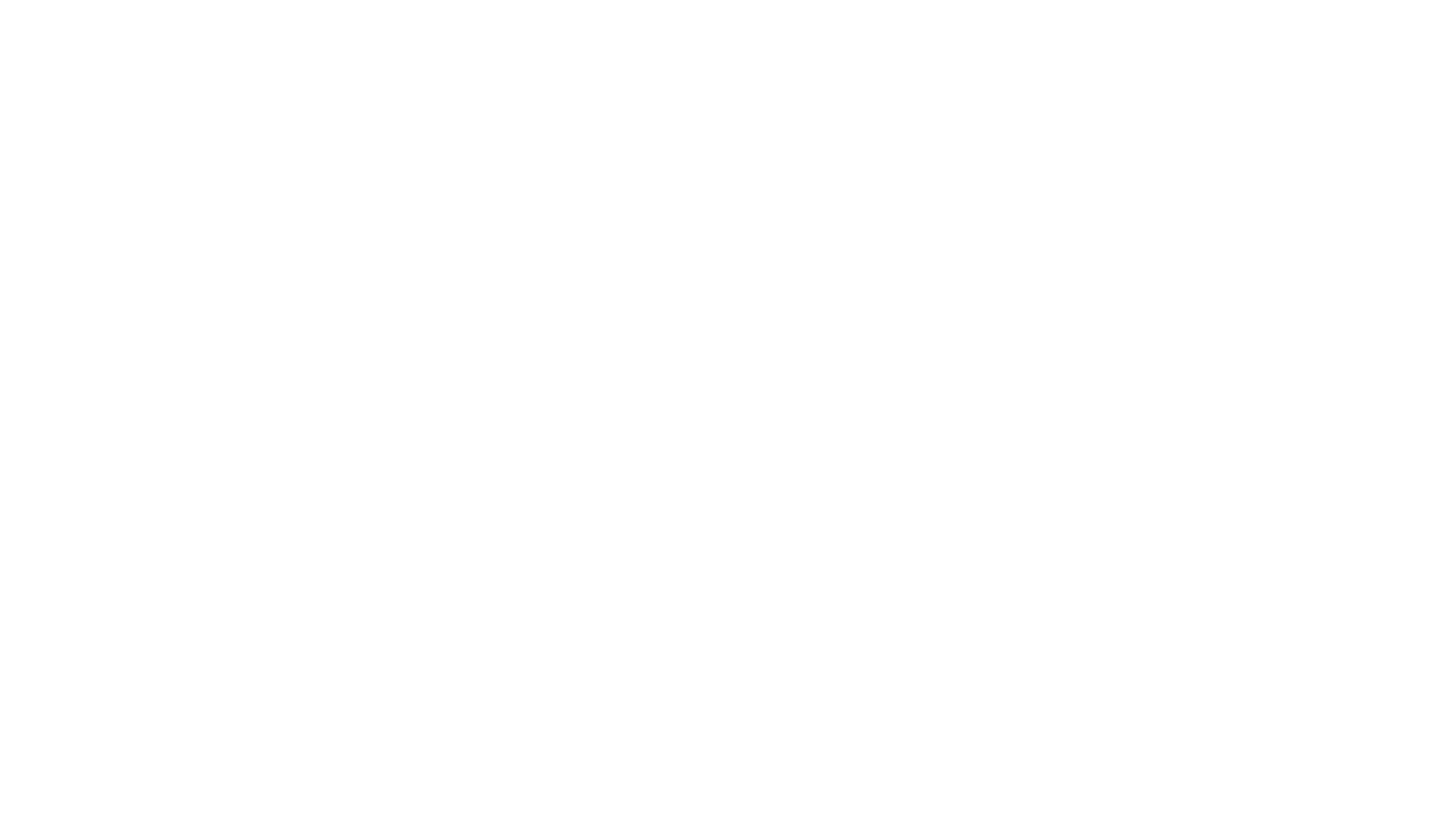

Supplement: Supplementary file 11 — Source Data [file 41467_2023_41054_MOESM11_ESM.zip › Source data/Supplementary Figure 7_The size-exclusion chromatography/Origin/Percentage/In summary.pptx]
